# Reference Values of Joint‐Specific Pressure Pain Thresholds in Healthy Male Individuals: A Retrospective Study

**DOI:** 10.1002/ejp.70050

**Published:** 2025-05-29

**Authors:** Fabian Tomschi, Alexander Schmidt, Marius Brühl, Thomas Hilberg

**Affiliations:** ^1^ Department of Sports Medicine University of Wuppertal Wuppertal Germany; ^2^ Department of Orthopaedics and Trauma Surgery University of Bonn Bonn Germany

**Keywords:** pain perception, pain sensitivity, pressure pain thresholds, reference values, somatosensory function

## Abstract

**Background:**

Pressure pain thresholds (PPT) are a component of Quantitative Sensory Testing and are used to assess mechanical pain sensitivity. Joint‐specific PPT measurements are relevant because many joint disorders involve altered pain processing at and around joints. This study aims to establish such reference values that might contribute to evaluate deviations in pain sensitivity in specific patient populations.

**Methods:**

This study retrospectively analysed PPT data from 407 healthy male individuals. Pressure was applied in a standardised manner using an algometer at eight anatomical landmarks: bilaterally at the elbow, knee and ankle joints, as well as the sternum and forehead. Percentile‐based, landmark‐discriminative normative data were calculated for PPT in general and segmented for age, BMI and mean subjective pain over 4 weeks.

**Results:**

Median (IQR) PPT values (N/cm^2^) for anatomical landmarks are as follows: ankle = 47.2 (35.9, 63.8), knee = 65.9 (48.6, 89.1), elbow = 58.0 (40.5, 81.1), sternum = 39.8 (30.2, 53.3) and forehead = 35.5 (26.8, 45.0). Between‐group analyses revealed no significant effect of BMI on PPT at any landmark assessed, no significant effect of age, except for the elbow joint (*p* = 0.035) and no effect of the presence of pain, except for the ankle (*p* = 0.020) and elbow joint (*p* = 0.010).

**Conclusion:**

This study provides normative joint‐specific PPT values in healthy male individuals, offering reference values for both clinical and research applications. These values can assist in interpreting PPT measured in clinical settings and in identifying abnormal pain sensitivity.

**Significance Statement:**

Pressure pain thresholds are an important measure in Quantitative Sensory Testing, yet comprehensive normative data for joint‐specific PPT in healthy individuals have been lacking. This study establishes reference values across multiple anatomical landmarks, providing a critical benchmark for evaluating pain sensitivity deviations in musculoskeletal disorders. These findings enhance the interpretation of PPT measurements, supporting improved pain assessment and diagnostic accuracy.

## 1 Introduction

Quantitative Sensory Testing (QST) is a standardised psychophysical method for evaluating the somatosensory system, including Aβ, Aδ and C fibre functioning, by measuring the responses to controlled thermal, mechanical or pressure stimuli (Mücke et al. 2021). The QST protocol includes the assessment of pressure pain thresholds (PPT) that provides information in understanding the patient's mechanical pain perception, pain sensitivity and related physiological responses in both clinical and research settings (Tomschi, Schmidt, et al. 2024; Vervullens et al. 2022). It allows for the identification of local and widespread mechanical hyperalgesia (Graven‐Nielsen and Arendt‐Nielsen 2010). Typically measured using pressure algometry, PPT provide a semi‐objective measure of mechanical pain sensitivity, which is defined as the point at which the applied pressure transitions from being perceived as an innocuous mechanical sensation to becoming painful (Vervullens et al. 2022). Unlike solely subjective measures of pain, such as visual analogue scales (VAS), numeric rating scales (NRS) or pain questionnaires, PPT offer a more objective assessment of pain sensitivity (Hjermstad et al. 2011; Robinson et al. 2024; Stausholm et al. 2023).

PPT are frequently employed in scientific studies, especially in patients with musculoskeletal joint diseases (e.g., knee pain (Sylwander et al. 2021), knee osteoarthritis (Arendt‐Nielsen et al. 2010; Kuni et al. 2015; Moss et al. 2016; Srimurugan Pratheep et al. 2018; Stausholm et al. 2023), rheumatoid arthritis (Bagnato et al. 2015; Catal et al. 2000; Hodge et al. 2009), haemophilic arthropathy (Ransmann et al. 2024; Schmidt et al. 2024), patellofemoral pain (Ferreira et al. 2022; Rathleff et al. 2013)), where mechanical pain sensitivity is often increased.

Mechanoreceptors and nociceptors in joint tissues respond to pressure stimuli during mechanical PPT measurements. Mechanoreceptors, primarily Aβ fibres, respond to mechanical stimuli, such as joint movement and stretch, and help regulate proprioception (Macefield 2021). Nociceptors, predominantly C‐fibres, remain silent under normal conditions (Oostinga et al. 2020) but become activated in response to noxious stimuli, such as mechanical distortion or local inflammation and acidosis (Vardeh et al. 2016). Additionally, Aδ fibres contribute to the transmission of sharp, immediate pain signals in response to mechanical and thermal stimuli. These receptors express various ion channels that can be sensitised under pathological conditions. In joint pathologies, such as osteoarthritis and rheumatoid arthritis, these responses can be altered due to inflammation and structural changes in joint tissues. Inflammatory mediators (e.g., pro‐inflammatory cytokines, chemokines, nerve growth factor, bradykinin, prostaglandins) sensitise nociceptors, lowering their activation threshold to, for instance, mechanical stimulation and causing exaggerated pain responses to normally non‐painful stimuli (allodynia) or heightened pain from painful stimuli (hyperalgesia) (Fang et al. 2023; Puntillo et al. 2021).

It is suggested that these somatosensory abnormalities can potentially be identified using PPT algometry (Stausholm et al. 2023) and PPT values could therefore be a useful diagnostic tool to identify possible signs of altered somatosensory processing, and thus to detect differences between healthy and patient populations (Vervullens et al. 2022). Additionally, joint PPT measurements can be used to monitor injury rehabilitation, for example, after anterior cruciate ligament reconstruction (Martin‐Alguacil et al. 2019) or inversion ankle sprain (Gogate et al. 2021), as well as in healthy individuals to explore, for instance, exercise‐induced hypoalgesia (Rice et al. 2019), where exercise can reduce mechanical joint pain sensitivity in healthy individuals (Tomschi, Schmidt, et al. 2024).

In 2006, the German Research Network on Neuropathic Pain (DFNS) published a protocol that aimed to standardise the positioning and procedures for evaluating QST including PPT (Rolke, Baron, et al. 2006). Within this protocol, reference values of 180 healthy individuals for three specific anatomical regions (i.e., the masseter muscle, the thenar eminence and the instep) were provided. Later, the DFNS published reference values of 162 healthy individuals for trunk landmarks that could be used as a reference for chronic back pain patients (Pfau et al. 2014). Besides, some publications present reference values of healthy individuals for body landmarks that are associated with, for instance, also low back pain (Suzuki et al. 2023) or migraine (Pan et al. 2024).

However, to the best of the authors' knowledge, there is a lack of comprehensive normative data on PPT values across ankle, knee and elbow joints in healthy populations. Given that these joints are frequently affected by the above‐mentioned musculoskeletal or neuropathic conditions, and as the knee and ankle are primary weight‐bearing joints subjected to mechanical stress, whereas the elbow is prone to repetitive strain injuries, normative joint PPT data are needed to understand deviations in PPT, as such values are the only way to estimate whether a patient presents altered somatosensory processing or not (Vervullens et al. 2022). Besides, additional measurements might help to detect central or widespread hyperalgesia, especially when using landmarks outside of the affected body regions (i.e., joints). Here, anatomical landmarks such as the sternum or forehead might provide additional information, as many MSK joint conditions, such as osteoarthritis, may involve central sensitisation (Arendt‐Nielsen et al. 2018), where the nervous system amplifies pain perception, leading to lower local and remote/central PPT values.

Based on these considerations, this study aims to provide reference values for joint‐specific PPT in a large sample of healthy male subjects and the following two research objectives are stated: (1) To provide joint‐specific reference values of PPT that were measured at uniform landmarks in a large sample and (2) to evaluate the impact of age, BMI and mild subjective pain on joint‐specific PPT.

## 2 Methods

### 2.1 Design and Study Population

Data presented herein were retrospectively collected from different individual original studies and analysed for the purposes of the study presented herein (see Figure S1 and Table S1). All original measurements were performed under quiet rest conditions before any type of intervention or as part of an observational design. Data of only male participants are included. These participants were included in the respective original studies all based on the same in‐ and exclusion criteria. These included that they were healthy and did not suffer from acute illness (e.g., flue) or acute injury (e.g., anterior cruciate ligament rupture) and any other general musculoskeletal disease (e.g., osteoarthritis, rheumatoid arthritis, fibromyalgia, chronic low back pain), further metabolic disease (e.g., diabetes mellitus), neuropathic pain syndromes (e.g., peripheral neuropathy, postherpetic neuralgia) or chronic headache and orofacial pain syndromes (e.g., chronic migraine). Participants were excluded when they reported a pain point prevalence (pain right now) or a 4‐week period prevalence (mean pain during the last 4 weeks) of NRS > 3. An NRS of ≤ 3 is indicative of no (0) or mild pain (1–3) (Nicholas et al. 2019). Acute (within 24 h pre‐measurement) or regular pain medication use also led to exclusion. Further, participants were asked not to have sore muscles. If a participant suffered from a minor local joint‐related injury (e.g., abrasion, bruise) at one joint, this participant was included but the respective joint was not considered in any analyses. Participants with psychiatric, motor or cognitive deficits or communication disorders that prevented participation were also excluded. All studies were approved by the local ethics committee and all participants provided written informed consent to participate in the respective study.

Given the retrospective nature of this study, no a priori sample size calculation was conducted. Participant inclusion focused on maximising the number of eligible individuals in accordance with predefined inclusion and exclusion criteria. This approach was employed to enhance the robustness of exploratory comparisons and to ensure adequate variability for subgroup analyses.

### 2.2 Data Collection and Measurements

All PPT measurements were performed by experienced personnel of the Department of Sports Medicine using a hand‐held digital pressure pain algometer (FPX 25 Compact Digital Algometer, Wagner Instruments, Greenwich, CT, USA). Each rater received a training of at least 6 h for the specific joint PPT measurements of the uniformly used landmarks (Reezigt et al. 2023), before a rater performed any study measurements to avoid inter‐rater discrepancies as much as possible. In general, PPT measurements using this specific device possess excellent intra‐rater (ICC = 0.92–0.96) and inter‐rater reliability (ICC = 0.94–0.96) in healthy participants (Koh et al. 2023).

**FIGURE 1:**
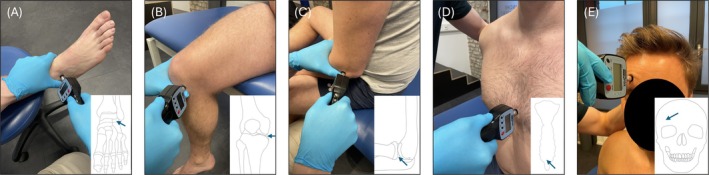
Pressure pain threshold (PPT) measurements performed at the landmarks ankle (A), knee (B) and elbow joints (C), as well as at the sternum (D) and forehead (E). Pictograms show a schematic anatomical visualisation where to administer the probe. All pictures and visualisations taken and created by the authors.

All measurements were performed using the same procedure at the same eight landmarks (Figure 1) after participants had rested for at least 5 min. In short, via the 1‐cm^2^ rubber tip of the algometer, pressure was applied to predefined landmarks: bilaterally at the elbow (lateral joint space below the lateral humeral epicondyle), knee (midpoint of the medial joint space below the medial femoral epicondyle), and ankle joints (lateral joint space between the lateral malleolus and talus bone) and the sternum (2 cm above its lower edge [xyphoid process]) as well as the forehead (1 cm above the midpoint of the right eyebrow [supraorbital margin]). Pressure was applied at a rate of 10 Newtons (N) per second. Before any study measurements, this procedure was explained thoroughly to the participants and at least one test measurement was performed in an exemplary manner at one landmark to familiarise participants with the procedure to avoid any confounding results due to the novelty of the measurement. Before each measurement, the participants were asked if they were ready. After receiving confirmation, the measurement was performed. Participants were instructed to say ‘stop’ when they first perceived the stimulus as painful. The measurement was then terminated and the force peak value was recorded in N. To prevent tissue damage, a cut‐off value of 140 N/cm^2^ was set in advance. The arithmetic mean resulting from three measurements was used for analysis, with a minimum pause of 10 s between each measurement.

### 2.3 Statistical Analyses

Anthropometric data are presented descriptively as mean ± standard deviation (range). Descriptive statistics for PPT are presented for each anatomical landmark, including the mean and standard deviation, as well as the median, minimum, maximum and percentile values (2.5th, 5th, 25th, 50th, 75th, 95th and 97.5th percentiles). To establish one value for the ankle, knee and elbow, the mean of the two respective joints was calculated and then further analysed. Age and BMI groups were established as follows: age: ≥ 18 – 29 (*n* = 212), ≥ 30 – 49 (*n* = 130) and ≥ 50 years (*n* = 65); BMI: normal < 25 (*n* = 210) and above normal ≥ 25 (*n* = 190). Furthermore, participants were grouped based on the pain status as having no pain (NRS = 0) and having mild pain (NRS = 1–3) for the pain point prevalence (*n* = 312 and *n* = 52, respectively) and the pain 4‐week period prevalence (*n* = 261 and *n* = 103, respectively). To analyse whether PPT differ significantly across BMI, age or pain status subgroups, Student's *t*‐tests or one‐way analyses of variance (ANOVA) were computed, respectively. Due to the partial presence of variance inhomogeneity or non‐normality of the data, values for PPT were log_10_‐transformed, as recommended (Rolke, Magerl, et al. 2006) and statistical analyses were performed using the log_10_‐transformed data, which can be found in Table S2. Results of PPT data are presented as the original non‐transformed value. Effect sizes were calculated and interpreted for Student's *t*‐tests (Cohen's *d*: small effect ≥ 0.2, medium effect ≥ 0.5 and large effect ≥ 0.8) and ANOVA (partial eta squared [$\eta_{\text{partial}}^{2}$]: small effect ≥ 0.01, medium effect ≥ 0.06 and large effect ≥ 0.14) according to conventional guidelines. Pearson correlation coefficients were computed and interpreted as follows: *r*: weak ≥ 0.1, moderate ≥ 0.3 and large ≥ 0.5 (Cohen 2013). Statistical analyses were conducted with SPSS version 29 (IBM, Armonk, NY, USA) with a universally set significance level of *α* ≤ 0.05.

## 3 Results

**FIGURE 2:**
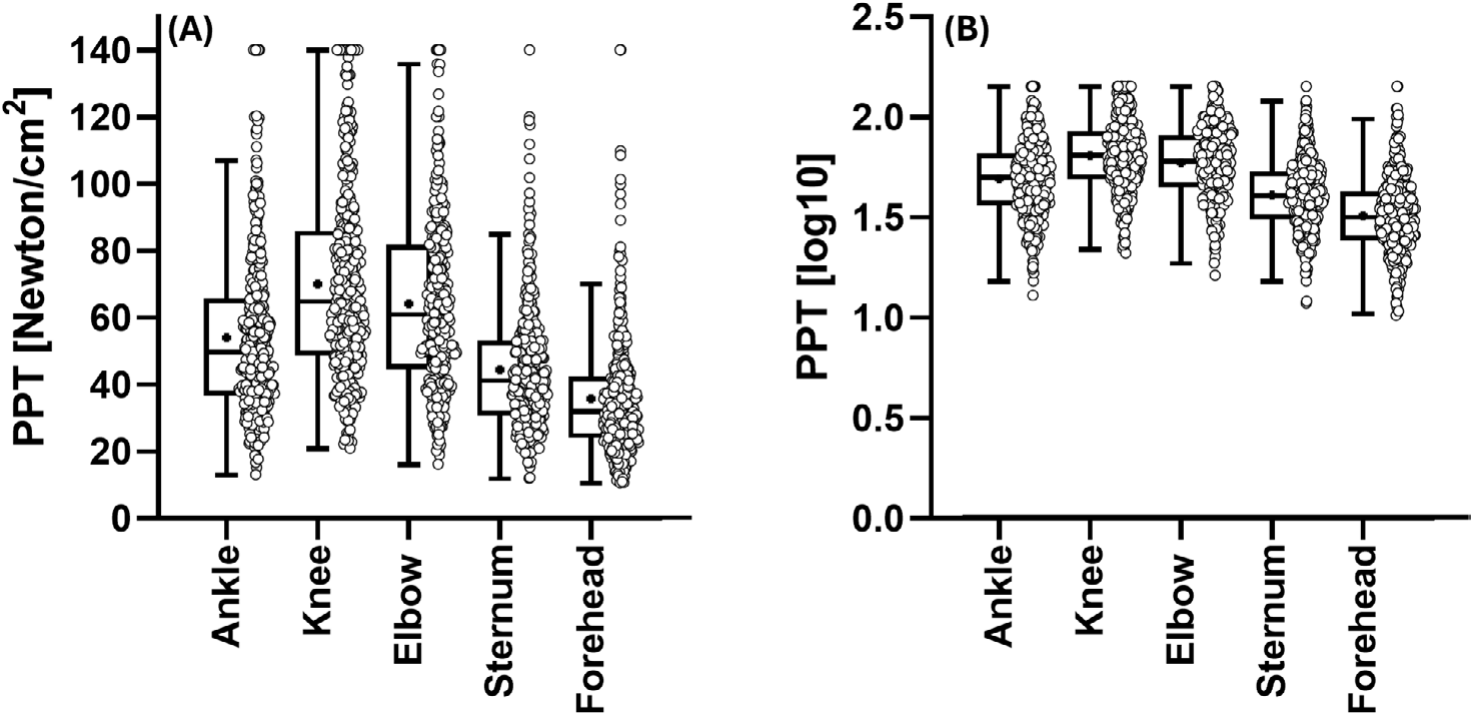
Boxplots and individual values of PPT presented as Newton/cm^2^ (A) and as log_10_‐transformed values (B). Boxes indicate data between 25th and 75th percentiles, with the horizontal bar representing the median and the cross representing the mean. Whiskers represent 1.5 × interquartile range.

PPT data of 407 healthy male participants were included (age [years]: 34.0 ± 12.8 (18.0–80.0); weight [kg]: 83.7 ± 10.9 (49.0–120.0); height [m]: 1.83 ± 0.07 (1.60–2.10); BMI [kg/m^2^]: 25.1 ± 3.0 [18.5–37.7]). PPT values for the entire sample are presented in Table 1 and Figure 2. The lower number of PPT values, especially of elbow joints, sternum and forehead, is due to the fact that in some of the original studies only lower extremity PPT were examined. In two participants, PPT were measured only at one knee and the other was excluded due to an abrasion in the area of knee PPT measurement. Further, in one participant, only one ankle was measured due to a non‐acute ankle sprain. PPT values differentiated according to age and BMI groups are presented in Tables 2 and 3, respectively. Here, the slightly lower numbers for the BMI datasets are explained by the fact that very little data (*n* = 7) were missing.

**TABLE 1:** Normative values of pressure pain thresholds (Newton/cm^2^) at the respective landmarks in healthy male individuals.

Landmark	Ankle	Ankle right	Ankle left	Knee	Knee right	Knee left	Elbow	Elbow right	Elbow left	Sternum	Forehead
*N*	407	406	407	407	406	406	339	339	339	377	397
Mean ± SD	54.1 ± 24.2	55.0 ± 25.4	53.1 ± 24.4	70.1 ± 28.2	69.8 ± 29.0	70.2 ± 29.1	64.2 ± 25.8	63.3 ± 26.3	65.0 ± 26.5	44.4 ± 19.0	35.8 ± 18.1
Median	49.7	49.5	47.5	64.8	64.4	65.0	60.9	58.7	61.4	41.2	31.9
Minimum	13.0	13.3	11.0	20.8	20.6	13.1	16.1	15.1	14.2	11.8	10.4
Maximum	140.0	140.0	140.0	140.0	140.0	140.0	140.0	140.0	140.0	140.0	140.0
2.5th percentile	21.3	21.2	19.7	26.8	26.9	25.9	24.7	23.2	24.3	19.7	13.1
5th percentile	24.1	23.6	23.4	31.5	31.0	30.3	28.4	28.0	28.1	21.1	15.6
25th percentile	36.7	36.5	35.9	48.7	47.9	49.3	44.7	42.8	44.5	30.9	24.1
50th percentile	49.7	49.5	47.5	64.8	64.4	65.0	60.9	58.7	61.4	41.2	31.9
75th percentile	65.8	69.4	64.6	86.0	86.2	86.5	81.8	82.9	80.7	53.2	42.6
95th percentile	100.4	106.0	102.5	131.3	135.2	138.2	112.2	111.0	117.8	80.3	70.2
97.5th percentile	119.5	119.9	120.2	140.0	140.0	140.0	124.3	123.1	130.7	93.7	81.5

**TABLE 2:** Normative values of pressure pain thresholds (Newton/cm^2^) at the respective landmarks in healthy male individuals stratified for age groups.

Age group	Landmark	Ankle	Knee	Elbow	Sternum	Forehead
18–29 years	*N*	212	212	183	204	209
	Mean ± SD	53.8 ± 23.2	68.4 ± 27.1	61.9 ± 25.9	43.9 ± 20.5	35.7 ± 17.3
	Median	47.7	64.6	56.6	39.4	31.9
	Minimum	21.2	20.8	20.1	12.0	10.5
	Maximum	140.0	140.0	140.0	120.0	108.5
	2.5th percentile	22.9	25.2	25.2	20.0	12.5
	5th percentile	25.1	30.7	27.4	21.8	14.9
	25th percentile	37.6	48.6	41.2	30.0	23.3
	50th percentile	47.7	64.6	56.6	39.4	31.9
	75th percentile	65.7	82.8	80.9	53.0	44.1
	95th percentile	102.2	122.7	112.1	84.7	76.1
	97.5th percentile	119.4	139.2	123.7	110.0	81.1
30–49 years	*N*	130	130	108	120	126
	Mean ± SD	52.9 ± 24.3	69.3 ± 27.0	64.0 ± 24.7	44.8 ± 17.6	35.4 ± 20.6
	Median	49.6	62.6	60.8	42.9	30.5
	Minimum	15.1	22.1	16.1	11.8	10.4
	Maximum	140.0	140.0	140.0	107.4	140.0
	2.5th percentile	17.6	26.6	24.0	17.9	14.5
	5th percentile	21.1	34.0	29.0	20.5	16.0
	25th percentile	35.3	49.3	45.7	31.8	23.3
	50th percentile	49.6	62.6	60.8	42.9	30.5
	75th percentile	64.3	85.2	80.9	54.4	39.4
	95th percentile	99.8	118.4	107.2	78.9	75.3
	97.5th percentile	118.8	140.0	124.7	89.8	99.3
≥ 50 years	*N*	65	65	48	53	62
	Mean ± SD	57.6 ± 27.3	77.1 ± 33.1	73.0 ± 26.6*	45.8 ± 16.0	36.6 ± 15.7
	Median	54.1	74.0	73.5	43.8	34.6
	Minimum	13.0	29.1	18.8	16.7	12.6
	Maximum	140.0	140.0	140.0	92.5	109.8
	2.5th percentile	15.3	29.2	19.0	17.6	12.8
	5th percentile	22.9	30.2	24.5	20.3	14.4
	25th percentile	36.0	49.6	59.0	35.8	26.8
	50th percentile	54.1	74.0	73.5	43.8	34.6
	75th percentile	76.9	99.9	87.9	52.9	44.4
	95th percentile	111.7	140.0	127.5	78.7	63.7
	97.5th percentile	140.0	140.0	138.6	87.7	85.9

* *p* ≤ 0.05 compared to young individuals (18–29 years), employing one‐way analysis of variance with Bonferroni adjustment.

**TABLE 3:** Normative values of pressure pain thresholds (Newton/cm^2^) at the respective landmarks in healthy male individuals stratified for BMI groups.

BMI group	Landmark	Ankle	Knee	Elbow	Sternum	Forehead
Normal weight (18.5–24.9 kg/m^2^)	*N*	210	210	171	196	206
	Mean ± SD	53.6 ± 24.2	72.2 ± 27.6	64.8 ± 26.7	43.5 ± 18.2	37.3 ± 19.6
	Median	48.1	67.4	58.7	40.2	32.9
	Minimum	15.1	20.8	18.8	12.0	10.4
	Maximum	140.0	140.0	140.0	120.0	140.0
	2.5th percentile	21.5	31.2	18.8	19.4	12.6
	5th percentile	24.5	34.4	29.0	20.8	14.9
	25th percentile	36.9	52.4	41.4	30.6	25.0
	50th percentile	48.1	67.4	58.7	40.2	32.9
	75th percentile	65.5	87.9	83.5	52.9	43.6
	95th percentile	105.9	130.9	117.4	78.3	78.7
	97.5th percentile	119.9	140.0	130.0	88.2	96.4
Above normal (≥ 25.0 kg/m^2^)	*N*	190	190	162	174	184
	Mean ± SD	54.7 ± 24.6	67.9 ± 29.1	63.8 ± 25.1	45.8 ± 20.0	34.2 ± 16.5
	Median	51.6	62.9	62.8	42.4	30.6
	Minimum	13.0	22.5	16.1	11.8	10.5
	Maximum	140.0	140.0	140.0	140.0	140.0
	2.5th percentile	20.7	25.2	24.5	19.8	13.8
	5th percentile	23.7	29.2	27.2	21.1	15.7
	25th percentile	36.5	46.7	45.5	31.6	23.4
	50th percentile	51.6	62.9	62.8	42.4	30.6
	75th percentile	67.1	85.2	81.1	53.5	41.0
	95th percentile	99.8	132.6	107.8	86.2	61.9
	97.5th percentile	116.7	140.0	126.3	100.1	78.1

ANOVA revealed a significant effect of age groups for PPT at the elbow (*p* = 0.041, $\eta_{\text{partial}}^{2}$ = 0.019), with 18‐ to 29‐year‐old individuals (61.9 ± 25.9) demonstrating lower PPT than individuals > 50 years of age (73.0 ± 26.6, *p* = 0.035). However, no significant effects were found for the ankle (*p* = 0.534, $\eta_{\text{partial}}^{2}$ = 0.003), knee (*p* = 0.215, $\eta_{\text{partial}}^{2}$ = 0.008), sternum (*p* = 0.484, $\eta_{\text{partial}}^{2}$ = 0.004) and forehead (*p* = 0.642, $\eta_{\text{partial}}^{2}$ = 0.002). Regarding BMI, no significant differences were observed between normal and above normal subgroups for any of the landmarks assessed (ankle: *p* = 0.720, *d* = −0.036; knee: *p* = 0.053, *d* = 0.194; elbow: *p* = 0.814, *d* = 0.026; sternum: *p* = 0.306, *d* = −0.107; forehead: *p* = 0.140, *d* = 0.150). No differences were observed between participants with no pain and mild pain based on the pain point prevalence at any landmark assessed (*p* > 0.05, *d* < 0.229). Significantly lower values were observed in participants with mild pain compared to those with no pain based on the pain 4‐week period prevalence at the ankle (*p* = 0.020, *d* = 0.273) and elbow (*p* = 0.010, *d* = 0.334) with no differences observed at the knee (*p* = 0.219, *d* = 0.143), sternum (*p* = 0.363, *d* = 0.110) and forehead (*p* = 0.420, *d* = 0.096). These data are presented in Table 4. In 43 participants, the two pain scales were asked verbally and it was then checked as not having pain without documentation of the exact value. These datasets were not considered in these analyses.

**TABLE 4:** Normative values of pressure pain thresholds (Newton/cm^2^) at the respective landmarks in healthy male individuals stratified for the average subjective pain intensity over the past 4 weeks on a numeric rating scale (NRS).

BMI group	Landmark	Ankle	Knee	Elbow	Sternum	Forehead
No pain (NRS = 0)	*N*	261	261	217	239	257
	Mean ± SD	55.3 ± 23.2	70.9 ± 28.8	66.3 ± 25.5	45.3 ± 19.6	35.7 ± 19.4
	Median	51.4	64.3	62.9	42.1	30.8
	Minimum	13.0	22.5	16.1	12.0	10.4
	Maximum	140.0	140.0	140.0	140.0	140.0
	2.5th percentile	22.6	31.2	25.2	17.8	13.0
	5th percentile	25.1	34.3	31.2	20.8	15.6
	25th percentile	38.5	49.1	46.8	31.5	23.6
	50th percentile	51.4	64.3	62.9	42.1	30.8
	75th percentile	67.1	86.4	84.9	52.9	42.3
	95th percentile	99.4	137.1	111.5	83.6	77.1
	97.5th percentile	117.5	140.0	124.5	97.2	93.4
Mild pain (NRS > 0 and NRS ≤ 3)	*N*	103	103	83	97	98
	Mean ± SD	50.2 ± 24.6*	67.3 ± 26.9	58.8 ± 26.3**	42.9 ± 17.6	36.5 ± 16.1
	Median	44.2	65.8	53.5	39.6	34.2
	Minimum	15.1	20.8	19.8	19.5	10.5
	Maximum	140.0	140.0	140.0	120.0	101.3
	2.5th percentile	20.5	24.1	20.2	20.6	12.4
	5th percentile	23.7	25.5	23.9	21.1	14.1
	25th percentile	32.2	48.2	39.2	29.8	26.5
	50th percentile	44.2	65.8	53.5	39.6	34.2
	75th percentile	58.0	83.3	80.0	53.8	44.0
	95th percentile	99.8	117.7	112.9	72.8	65.9
	97.5th percentile	128.0	127.1	134.1	90.3	83.5

*p ≤ 0.05, **p ≤ 0.01 compared to individuals with no pain (NRS = 0), employing the Student's *t*‐tests for unpaired samples.

**FIGURE 3:**
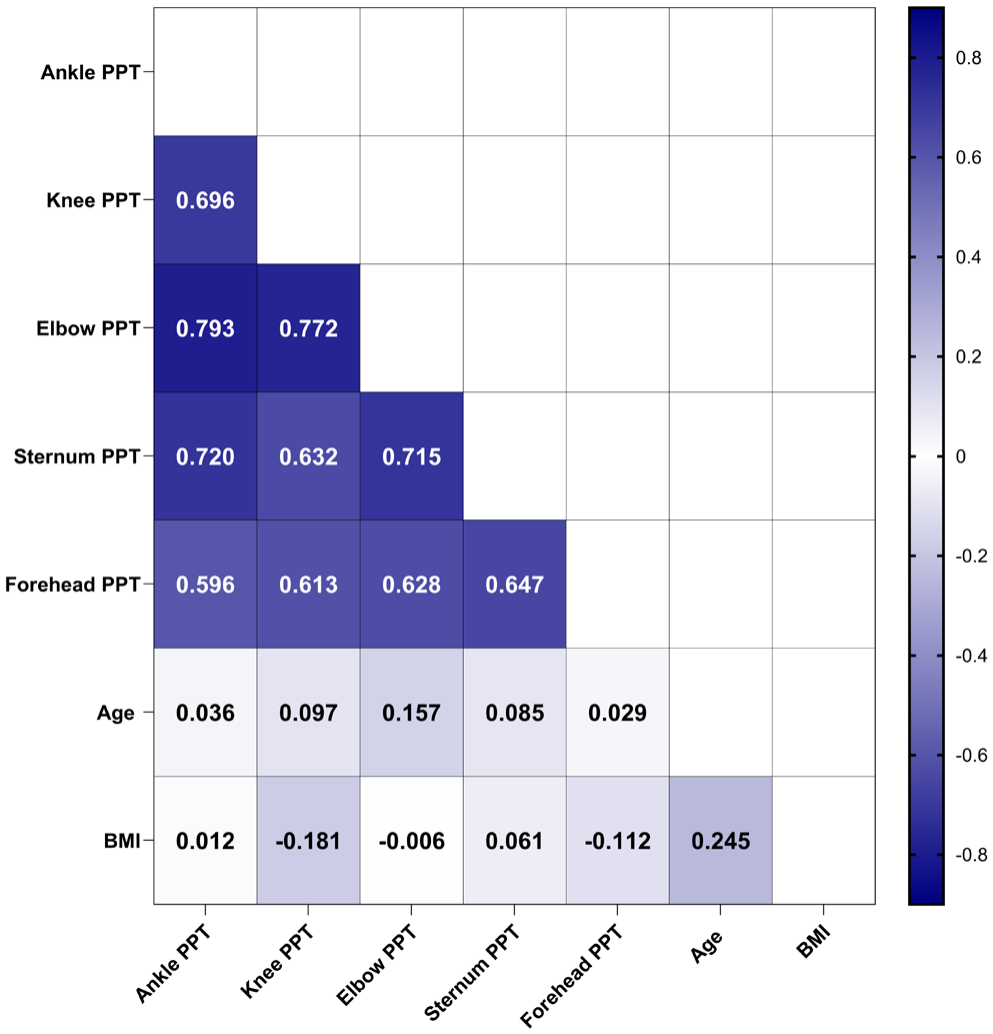
Correlogram for correlations between the respective pressure pain thresholds (PPT) with age and BMI. Results are presented as *r* values. The darker the colour of the boxes the stronger the correlation is (see bar right to the correlogram).

Lastly, Pearson correlation analyses between different pooled landmarks as well as age and BMI are presented in Figure 3. Results of the side‐by‐side comparisons of the same landmark revealed that PPT for each landmark were significantly positively inter‐correlated. Large effect sizes with the left and right body sites for the ankle (*r* = 0.893, *p* < 0.001), knee (*r* = 0.884, *p* < 0.001) and elbow (*r* = 0.939, *p* < 0.001) are observed. Additionally, PPT at the knee were significantly inversely correlated with BMI (*r* = −0.181, *p* < 0.001), and PPT at the elbow significantly correlated with age (*r* = 0.157, *p* = 0.004) albeit both showing weak coefficients.

## 4 Discussion

This study presents normative joint‐specific PPT values for healthy male individuals. Although PPT are widely used as a measure to assess pain perception in different cohorts, comprehensive data on what constitutes normal joint PPT values in healthy male individuals are not available. Previous work by the DFNS established normative PPT values for regions such as the masseter muscle, thenar eminence, instep and trunk landmarks (Pfau et al. 2014; Rolke, Baron, et al. 2006). More recently, additional reference values have been provided for pain‐associated sites like, for instance, the lower back and head (Pan et al. 2024; Suzuki et al. 2023). However, joint‐specific reference data for healthy male individuals remain unavailable. This study aimed to fill this gap by providing data for joint and non‐joint landmarks, that is, the ankle, knee, elbow as well as for the sternum and forehead that can serve as a reference for future research and clinical applications.

Previous research on joint PPT has demonstrated that PPT are, in general, lower in patients with musculoskeletal joint diseases compared to healthy individuals, reflecting increased pressure pain sensitivity and local hyperalgesia. For instance, within individuals suffering from current knee pain with no diagnosed osteoarthritis, no rheumatologic disorder or cruciate ligament injury, Sylwander et al. (2021) observed median knee PPT of 32.4 N/cm^2^ measured at similar landmarks as presented herein. Based on the present reference values, these PPT values are to be seen slightly above the 5th percentile. In a test–retest design, Srimurugan Pratheep et al. found knee PPT of 32.4 and 28.8 N/cm^2^ in patients suffering from knee osteoarthritis. Again, these values can be seen slightly above the 5th percentile.

PPT values of this study present a large inter‐person variability (see Figure 2) and various factors can modulate an individual's pain perception. In a fairly recent and comprehensive article, Fillingim (2017) highlighted several demographic factors, including age, sex and ethnicity, alongside different biopsychosocial factors, such as genetic predisposition and psychological disorders, as potential contributors to the inter‐individual variance in experimentally induced pain perception. Regarding the influence of age, the author concludes that PPT values are generally increasing with age, which is consistent with other reviews regarding this topic (Lautenbacher 2012). Contrastingly, a recent meta‐analysis reported PPT to significantly decrease with advancing age (El Tumi et al. 2017). In the present study, interestingly, no significant differences in PPT were observed between age groups either, except for the elbow joint. However, a descriptive comparison between younger (18–29 years) and older individuals (> 50 years) revealed that older people consistently exhibit higher PPT values, especially at articular landmarks. In the present study, individuals with diagnosed joint or musculoskeletal disease were excluded. Yet, it remains elusive whether differences in pain perception and pain processing are primarily a result from age‐related alternations on a neuro‐physiological level (González‐Roldán et al. 2020; Zhi et al. 2024) or a result from subclinical and undiagnosed age‐related joint alterations (Donat et al. 2005) that contribute to potential differences in pain perception of different age groups. As chronological age represents only one of many potential factors that might modulate someone's pain perception, the heterogenic results across studies could be explained by uneven distribution of other, more impactful variables. These should therefore be considered and controlled for in upcoming investigations to generate more robust estimates.

Regarding BMI, results indicate no significant differences between individuals with normal (18.5–24.9 kg/m^2^) or above normal (> 25 kg/m^2^) values. Research associated with body composition has not consistently shown to be significant predictors of an individual's experimentally induced pain perception. While some studies found BMI or body fat percentage to be significantly associated with PPT (Dioguardi et al. 2019; Sufiyan et al. 2023; Tashani et al. 2017), others did not (Fedders et al. 2019; Mariani et al. 2020). These contradicting findings might, in part, result from heterogeneity in the measurement site assessed. According to a study by Kosek et al. (1999), PPT values differ significantly depending on the specific anatomical landmark examined. The results presented in this study are based exclusively on bony articular landmarks. However, the selection of the presented anatomical joint landmarks can be particularly valuable for ensuring reliable and consistent PPT measurements over time for longitudinal or even independent studies. These joint landmarks provide stable reference points that reduce variability between measurements. These fixed points minimise the influence of muscle‐related factors, such as fatigue or soreness, that might otherwise affect PPT readings. This is important, as muscle fatigue or soreness represent potential confounders, especially in musculoskeletal research (Abboud et al. 2019; Fleckenstein et al. 2017; Pearcey et al. 2015).

With respect to the subjective pain state, results show that PPT did not differ between individuals reporting no pain and those with mild pain based on point prevalence, suggesting that momentary mild pain does not significantly affect mechanical pain sensitivity. However, when considering the 4‐week period prevalence, participants with mild pain demonstrated significantly lower PPT at the ankle and elbow compared to pain‐free individuals, albeit with low effect sizes. This might be indicative of higher localised mechanical pain sensitivity in these joints, whereas no significant differences were observed at the knee, sternum or forehead. These findings might suggest that even mild, recurrent pain over a period of time (e.g., 4 weeks) may be associated with increased general or localised sensitivity at specific joint sites, even though the correlation between PPT and subjective pain measures is generally low (e.g., Sanches et al. 2015).

Finally, correlation analyses for PPT between different landmarks and bilateral similar body parts consistently showed strong effects (*r* ≥ 0.5). This finding is in line with previous research in healthy individuals. For instance, Bhalang et al. (2005) reported significant correlations between PPT at the temporalis muscle, the masseter muscle and temporomandibular joint (*r* = 0.66–0.70). Similarly, Lacourt et al. (2012) stated that the highest degree of convergent validity was associated with side‐by‐side PPT comparisons with a Cronbach's alpha value above 0.89. In the present study, strong correlations for PPT were found between various and within bilaterally assessed bony landmarks. These findings indicate that PPT measurements are highly correlated across different body regions, regardless of the tissue type. A particularly noteworthy and potentially clinically relevant observation is the high consistency of PPT values in side‐to‐side comparisons among healthy individuals. This tendency suggests that, in cases of asymmetrical musculoskeletal impairment due to, for instance, inflammatory or degenerative joint processes, PPT values may increasingly diverge between the two sides of the body.

Apart from the clinical application, joint PPT were also employed in the context of exercise‐induced hypoalgesia. For instance, it was shown that a maximal intensive 90‐s isokinetic bicycle exercise increases PPT of the left and right ankle from 70.0 and 66.6 (pre‐exercise) to 95.7 and 90.8 N/cm^2^ (post‐exercise), respectively, hence inducing hypoalgesia in healthy subjects (Tomschi, Schulz, et al. 2024). Post‐exercise values are comparable with those observed in healthy individuals within the upper decile. These values can potentially be used to explore and interpret acute changes as well as changes resulting from long‐term interventions in pain sensitivity (Belavy et al. 2021).

### 4.1 Strengths and Limitations

This study for the first time presents joint‐specific PPT reference values based on a large sample of healthy male individuals (*n* > 400) using the same methodology and the same predefined landmarks, which were assessed at the same department by experienced and uniformly instructed researchers. This dataset provides normative values for joint‐specific PPT, which can be applied in both clinical and research settings to assess deviations in pressure pain sensitivity and somatosensory function. Yet, there are several limitations that need to be acknowledged to adequately interpret and use these data. First and most importantly, the study sample consisted solely of male participants, limiting the generalisability of the findings to females. Future research is warranted to expand the data presented to female individuals. This study is to be seen as a retrospective analysis of data derived from different studies and was therefore not prospectively registered. The study's focus was on providing data of healthy individuals on joint PPT and does not present data of specific patient groups. Other anatomical landmarks that are affected by prevalent musculoskeletal diseases like for instance low back pain, neck pain or hip osteoarthritis are not considered. In the present study, no PPT data for muscular or extra‐articular landmarks such as tendons are presented, which limits its applicability for evaluating pain sensitivity in non‐articular regions. The lack of data of further influencing factors such as for example physical activity profile, sleep quality, psycho‐social factors, which are known to influence pain perception (Brellenthin et al. 2017; Lee et al. 2024; Xu et al. 2022) represents another limitation. A further potential limitation is the use of a relatively fast pressure application rate (10 N/s) during PPT assessment, which may have limited participants' reaction time and potentially introduced over‐ or underestimation of actual PPT, especially in participants with a high pressure pain sensitivity. Yet, all measurements started after the participant confirmed that he is ready.

## 5 Conclusion

To conclude, the presentation of these joint PPT reference values of healthy males possesses a clinical relevance as these values can help to identify and interpret abnormal pain sensitivity in patients. Only PPT values of the elbow seem to be higher in older adults, whereas PPT values do not seem to be dependent on BMI. Yet, participants with mild pain (NRS of 1–3) show a higher mechanical pain sensitivity at the ankles and elbows compared to those with no pain. The presented values might be useful to assess altered local mechanical pain sensitivity or for tracking the effectiveness of interventions, such as physical therapy or pharmacological treatments, by providing a reference for PPT measurements.

## Author Contributions

Fabian Tomschi and Thomas Hilberg had the initial idea of the study. Fabian Tomschi and Alexander Schmidt designed the study. Marius Brühl, Alexander Schmidt and Fabian Tomschi performed data curation. Fabian Tomschi and Alexander Schmidt analysed the results. Fabian Tomschi and Alexander Schmidt wrote the first draft of the paper. Thomas Hilberg supervised the study. Thomas Hilberg and Marius Brühl edited the manuscript and provided intellectual content.

## Conflicts of Interest

The authors declare no conflicts of interest.

## Supporting information


Figure S1

Table S1

Table S2

## Data Availability

The data that support the findings of this study are available from the corresponding author upon reasonable request.
