# Supplementary figures and images for: Reference Values of Joint‐Specific Pressure Pain Thresholds in Healthy Male Individuals: A Retrospective Study
Source: Eur J Pain. 2025 May 29;29(6):e70050. doi: 10.1002/ejp.70050 (PMC12123253; doi:10.1002/ejp.70050)

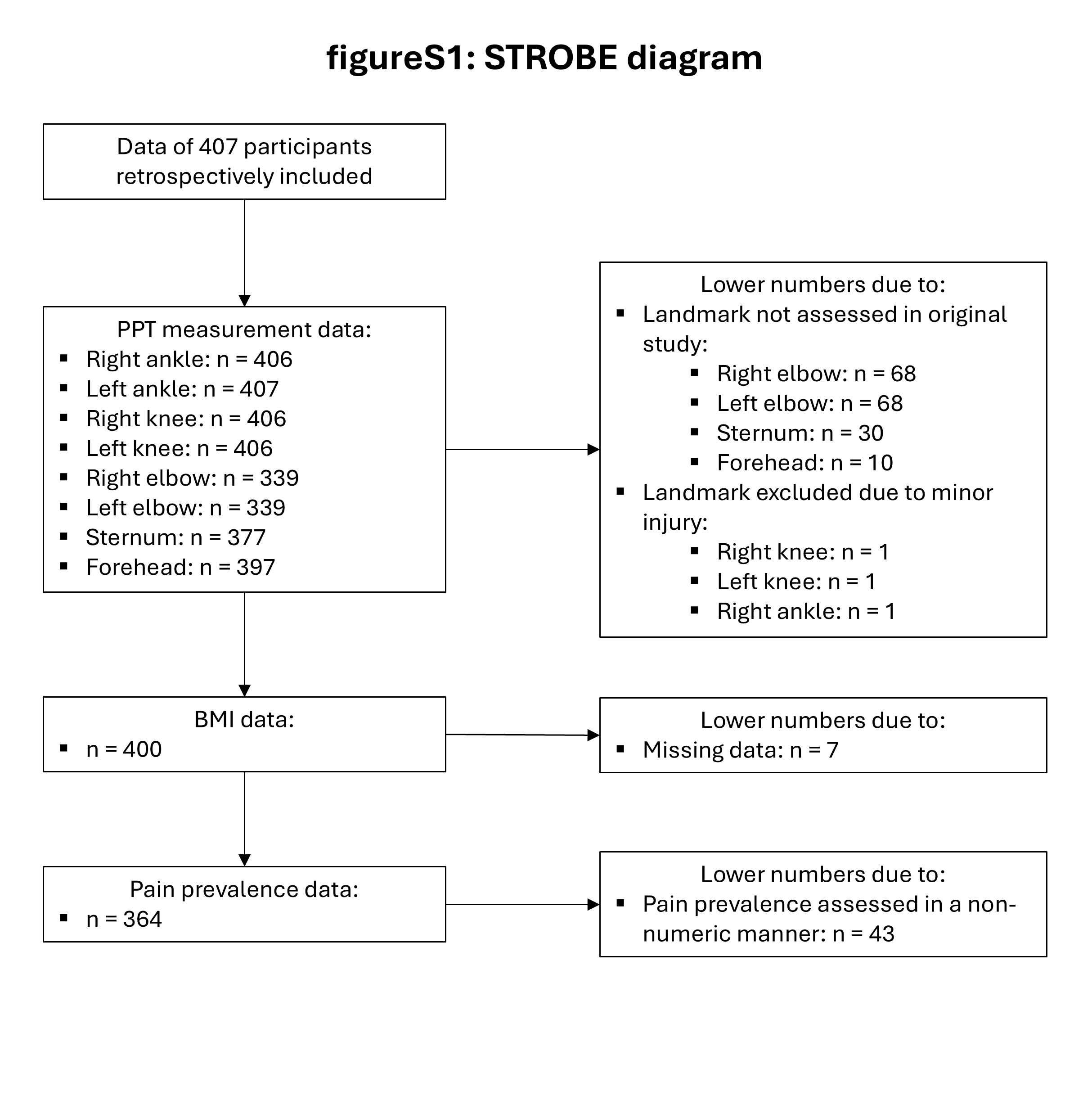

Supplement: Supplementary file 1 — Figure S1 [file EJP-29-0-s001.tif]
