# Supplementary material for: Reference Values of Joint‐Specific Pressure Pain Thresholds in Healthy Male Individuals: A Retrospective Study
Source: Eur J Pain. 2025 May 29;29(6):e70050. doi: 10.1002/ejp.70050 (PMC12123253; doi:10.1002/ejp.70050)
Supplement: Supplementary file 3 — Table S2 [file EJP-29-0-s003.docx]

**TableS2:** Normative values of log10-transformed pressure pain thresholds [Newton/cm^2^] at the respective landmarks

| **Landmark** | **Ankle** | **Ankle right** | **Ankle left** | **Knee** | **Knee right** | **Knee**  **left** | **Elbow** | **Elbow right** | **Elbow left** | **Sternum** | **Forehead** |
| --- | --- | --- | --- | --- | --- | --- | --- | --- | --- | --- | --- |
| N | 407 | 406 | 407 | 407 | 406 | 406 | 339 | 339 | 339 | 377 | 397 |
| Mean ± SD | 1.69 ± 0.19 | 1.70 ± 0.20 | 1.68 ± 0.19 | 1.81 ± 0.18 | 1.81 ± 0.18 | 1.81 ± 0.19 | 1.77 ± 0.18 | 1.76 ± 0.19 | 1.77 ± 0.18 | 1.61 ± 0.18 | 1.51 ± 0.19 |
| Median | 1.70 | 1.69 | 1.68 | 1.81 | 1.81 | 1.81 | 1.78 | 1.77 | 1.79 | 1.61 | 1.50 |
| Minimum | 1.04 | 1.11 | 1.04 | 1.31 | 1.31 | 1.31 | 1.18 | 1.18 | 1.18 | 1.07 | 1.01 |
| Maximum | 2.15 | 2.15 | 2.15 | 2.15 | 2.15 | 2.15 | 2.15 | 2.15 | 2.15 | 2.15 | 2.15 |
| 2.5th percentile | 1.33 | 1.33 | 1.30 | 1.43 | 1.43 | 1.41 | 1.39 | 1.36 | 1.39 | 1.29 | 1.12 |
| 5th percentile | 1.38 | 1.37 | 1.37 | 1.50 | 1.49 | 1.48 | 1.45 | 1.45 | 1.45 | 1.32 | 1.19 |
| 25th percentile | 1.56 | 1.56 | 1.56 | 1.69 | 1.68 | 1.69 | 1.65 | 1.63 | 1.65 | 1.49 | 1.38 |
| 50th percentile | 1.70 | 1.69 | 1.68 | 1.81 | 1.81 | 1.81 | 1.78 | 1.77 | 1.79 | 1.61 | 1.50 |
| 75th percentile | 1.82 | 1.84 | 1.81 | 1.93 | 1.94 | 1.94 | 1.91 | 1.92 | 1.91 | 1.73 | 1.63 |
| 95th percentile | 2.00 | 2.03 | 2.01 | 2.12 | 2.13 | 2.14 | 2.05 | 2.05 | 2.06 | 1.90 | 1.85 |
| 97.5th percentile | 2.08 | 2.08 | 2.08 | 2.15 | 2.15 | 2.15 | 2.09 | 2.09 | 2.11 | 1.97 | 1.91 |
